# Characteristics of Non-Emergent Visits in Emergency Departments: Profiles and Longitudinal Pattern Changes in Taiwan, 2000–2010

**DOI:** 10.3390/ijerph16111999

**Published:** 2019-06-05

**Authors:** Liang-Chung Huang, Wu-Fu Chung, Shih-Wei Liu, Jau-Ching Wu, Li-Fu Chen, Yu-Chun Chen

**Affiliations:** 1 Department of Emergency Medicine, National Yang-Ming University Hospital, I-Lan 26042, Taiwan; horus7855@yahoo.com.tw (L.-C.H.); wolfchung2001@yahoo.com.tw (W.-F.C.); shihweiliu123@gmail.com (S.-W.L.); 2 School of Medicine, National Yang-Ming University, Taipei 11221, Taiwan; jauching@gmail.com; 3 Department of Neurosurgery, Taipei Veterans General Hospital, Taipei 11217, Taiwan; 4 Department of Family Medicine, Taipei Veterans General Hospital, Taipei 11217, Taiwan; 5 Institute of Hospital and Health Care Administration, National Yang-Ming University, Taipei 11221, Taiwan

**Keywords:** emergent department (ED), non-emergent ED visits, cohort effect, health utilization

## Abstract

An increasing number of emergency department (ED) visits have posed a challenge to health systems in many countries, but an understanding of non-emergent ED visits has remained limited and contentious. This retrospective study analyzed ED visits using three representative cohorts from routine data to explore the profiles and longitudinal pattern changes of non-emergent ED visits in Taiwan. Systematic-, personal-, and ED visit-level data were analyzed using a logistic regression model. Average marginal effects were calculated to compare the effects of each factor. The annual ED visit rate increased up to 261.3 per 1000 population in 2010, and a significant one-third of visits were considered as non-emergent. The rapidly growing utilization of ED visits underwent a watershed change after cost-sharing payments between patients and medical institutions were increased in 2005. In addition to cohort effects resulting from cost-sharing payment changes, all factors were significantly associated with non-emergent ED visits with different levels of impact. We concluded that non-emergent ED visits were associated with multifaceted factors, but the change to cost-sharing payment, being female, younger age, and geographical residence were the most predictive factors. This information would enhance the implementation of evidence-based strategies to optimize ED use.

## 1. Introduction

The rapidly increasing numbers of emergency department (ED) visits has posed a challenge to health systems in many countries [1,2,3]. Many studies assessing the appropriateness of ED use have found that a large portion of ED visits were considered non-emergent—those conditions for which a delay in treatment or treatment in another care site (e.g., outpatient office or clinic) would not increase the likelihood of an adverse outcome [4,5,6,7]. Although the non-emergent ED visits utilization rates vary by country, it is believed that they may lead to excessive healthcare spending, unnecessary testing and treatment, and represent a missed opportunity for longitudinal relationships with primary care physicians.

Many factors contribute to increasing rates of non-emergent use of ED visits. Previous studies suggested quite a few factors that can be divided into systematic-, personal-, and visit-level factors, such as cost-sharing policy, ageing population, chronic disease profile of the population, personal health system utilization, personal socioeconomic influences, and availability of ED service [1,7,8,9,10,11]. With the introduction of a routinely collected database which provides every detail of information at different levels, it is suggested that non-emergent use of ED visit should be considered by capita or even by visit [9,12,13,14] for a more sophisticated strategy for ED resources optimization [15]. 

It is crucial to identify factors for non-emergent ED visits using real world data. Taiwan’s health system has made itself one of the most extensive fields of study for population health [16]. The national health insurance system in Taiwan features a high coverage rate of 99% of the entire 23 million population and enables unrestricted access to healthcare providers. Patients are allowed to choose either ED or an outpatient department service at any level (ranging from clinics to medical centers) in Taiwan. Moreover, the introduction of increased cost-sharing payments for ED visits may also have influenced non-emergent ED use. Using such real world data may provide us a better understanding of the patients’ decision process to regard to non-emergent ED visits through their utilization pattern.

The present study aimed to examine the factors associated with non-emergent ED visits. Using the real world data, systematic-, personal- and visit-level factors previously thought to affect non-emergent ED visits separately were considered together in this study. The results should add unique information for evidence-based decision making to optimize ED use.

## 2. Materials and Methods 

### 2.1. Data Source and Ethical Concerns

In this study, we incorporated three representative cohorts from routine data to explore the profiles and longitudinal changes of non-emergent ED visits in Taiwan. Taiwan’s Government implemented a National Health Insurance (NHI) program in 1995. Taiwan’s NHI program covers 99% of the Taiwanese population and is contracted with 97% of the providers of healthcare services in Taiwan, providing unrestricted access to medical care and universal health insurance for all residents in Taiwan. For study purpose, the National Health Research Institutes had recompiled these data into a longitudinal research dataset (Longitudinal Health Insurance Database, LHID), with a vigorous encryption de-identification and anonymization process, which is randomly sampled from the whole population every five years. Each LHID contains all the original claim data of one million beneficiaries enrolled in the sample year and named after the year of sampling. Currently, there are three datasets (LHID2000, LHID2005, and LHID2010, respectively) available to the public. There is no significant difference in the gender distribution between the patients in the LHID and the entire population in Taiwan (https://nhird.nhri.org.tw/en/Data_Subsets.html).

This study used the above datasets as the data source. These datasets contained comprehensive information on the insured subjects, including gender, date of birth, dates of clinical visits (both preventive services and emergent visits), the International Classification of Diseases (Ninth Revision) Clinical Modification (ICD-9-CM) codes of diagnoses, expenditure amounts, and characteristics of health providers (such as geolocation and accreditation levels). 

This study was initiated after approval from the Institutional Review Board of the National Yang-Ming University Hospital, Taiwan (NYMUH IRB No. 2014A020) and the National Yang-Ming University, Taiwan. The Institutional Review Board waived the requirement for written informed consent from each of the patients involved since all identifying personal information in the LHID is encrypted.

### 2.2. Determining of Non-Emergent ED Visit

We used the NYU algorithm developed by the Center for Health and Public Service Research of New York University to classify ED utilization [17]. The NYU algorithm was developed in 2000 and was aimed at assessing the level of diagnosed severity associated with ED visits. ED visits were classified into the following severity categories: “Non-emergent (NE)”, “Emergent, but primary care treatable (EDPCT)”, “Emergent, preventable or avoidable (EDPA)” and “Emergent, not preventable (EDNPA)”. The NYU algorithm provides the probability that each ED visit primary diagnosis code falls into one of the aforementioned four severity categories. The NYU algorithm has been well validated in the United States as well as in Taiwan, showing a high correlation with subsequent readmission and mortality [18,19].

The current study used the NYU algorithm to classify the severity of ED visits. Each of the enrolled ED visits was classified as either “Non-emergent visit” or “Emergent visit” according to the probability for severity categories. The summation of probability for NE and EDPCT greater than or equal to a probability threshold of 0.75 (i.e., NE + EDPCT ≥ 0.75) was defined as “Non-emergent visit”, whereas the rest of ED visits were deemed as “Emergent visits.” To increase accuracy, we also adopted the updated version of the NYU algorithm [20]. To compare “Non-emergent visits” and “Emergent visits,” we excluded conditions such as injuries, psychosis, and alcohol or drug problems from our regression model since these conditions usually require ED services regardless of severity.

### 2.3. Systematic-Level Factor and Study Cohort

In this study, we used ED visits made by three cohorts at three time points: 2000, 2005, and 2010. We enrolled all ED visits made between January 1 and December 31 in each of the years 2000, 2005, and 2010. For each ED visit, we extracted each patient’s record for their past history of health utilization (up to 1 year) and comorbidities (up to 2 years). Such three-waved, cross-sectional design enabled us to compare the pattern of changes over time and policy changes.

There is a systematic change for the introduction of a 10–20% increase in copayment in 2005 trying to cut down the rapid growth of ED visit. In Taiwan, patients were required to pay a fixed amount of cost-sharing payments for their ED visits unless waived in particular scenarios. The cost-sharing payments varied by hospital level, i.e., the cost-sharing payment for ED visits in medical centers, metropolitan hospitals, and local community hospitals were at a fixed amount premium of NTD$420, NTD$210, and NTD$120, respectively. To control the rapid increase in ED visits, the cost-sharing was raised in 2005, i.e., the cost-sharing payment for medical centers, metropolitan hospitals, and local community hospital increased to NTD$450, NT$300, and NTD$150, respectively. 

Since Taiwan’s ED utilization pattern was affected greatly by copayment change [21,22], the current three-waved study design provided an extraordinary opportunity to quantify the influence resulting from the change of the cost-sharing payment. As a result, we included cohort year as a systematic-level factor in the following regression model to account for system change, i.e., year 2000 reprented the ED visit pattern under the original copayment plan (five years earlier), the year 2005 ED visit pattern represented the introductory year of the increased copayment plan, while year 2010 represented the ED visit pattern under the increased copayment plan (five years later).

### 2.4. Personal- and ED Visit-Level Factors

We collected both personal- and visit-level factors including personal demographics, socioeconomic status, past health utilization, and characteristics of ED visits to assess factors associated with non-emergent visits. Subjects’ co-morbidities were categorized according to Elixhauser’s comorbidity model by the presence of either diagnostic codes in the outpatient records or discharge codes in the database within two years before the date of the visit [10]. The patient’s income level was determined by the monthly income they reported to the National Health Insurance Administration. Patients who were unemployed and enrolled as a dependent through their relative (i.e., parents, spouses, or children) were categorized as dependent. The geography and urbanization level of their living area was determined by NHI registration data.

Each patient was tracked back for 365 days for their health utilization pattern in terms of hospitalization, ED visits, outpatient visits, and Traditional Chinese Medicine (TCM) use. Because all medical care providers are contracted with the NHI, which allows all patients unrestricted access, any admission, ED visit, or outpatient visit would be tracked, even if they were in different institutes or geographically distant. As a result, there was little loss to follow-up, and the recall bias for health utilization would be minimal.

The characteristics of visits, including the hospital accreditation level and visit date were extracted from the database. Seasons, days of the week, and public holidays were further determined for the visit dates. In Taiwan, public holidays including weekends are usually nonworking days and most outpatient clinics are closed on those days during the year. For patients who have mild illness on holidays, some of them will head to ED directly, while some of them will wait for outpatient clinics on the next working day. As a result, emergency departments are often more crowded with non-emergent and emergent patients on public holidays than on working days.

Cost-sharing payment plays a controversial role in non-emergent ED visits [12,13]. In Taiwan’s NHI scheme, cost-sharing payment is waived for children less than three years old, older adults aged more than 100 years, and for severe illness, and we included this variable in the current analysis.

### 2.5. Statistical Analysis

All of the data were linked using the SQL server 2017 (Microsoft Corp, Redmond, WA, USA) and analyzed by Stata software (Stata Corporation, College Station, TX, USA). The annual ED visits were calculated to demonstrate the change of usage pattern of ED in the study cohort. A logistic regression model was used to assess risk factors associated with non-emergent ED visits. An adjusted odds ratio (AOR) for non-emergent ED visits for each factor was estimated by controlling other factors in the model. To quantify the effect of levels of each factor, average marginal effect (AME) of factors was calculated, which is the average predicted probabilities that would be observed if the whole study population were in the same level of each factor with all else being left as it was in the data. AME were expressed as a percentage, and were interpreted as the average predicted probabilities of being non-emergent ED visits if they were average in such a level of factors. The most frequent levels of each factor were used as the base value. A two-tailed level of 0.05 was considered statistically significant.

## 3. Results

### 3.1. Annual ED Visit and Proportion of ED Visit

**Figure 1:**
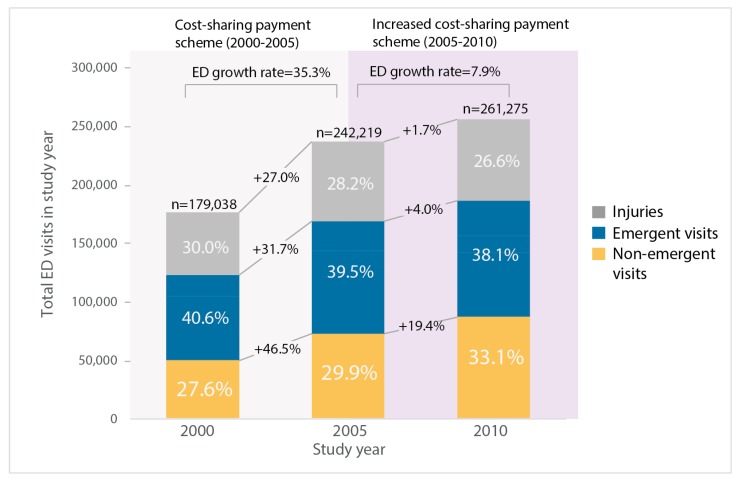
Annual emergency department (ED) visit, growth rates, and proportions of ED visit classification by study year from three representative study cohorts (LHID2000, LHID2005, LHID2010) in Taiwan. (*n* = 1,000,000 for each cohort). Growth rates of ED visits are expressed in percentages and overlaid on lines between study years. Background shading in different colors represents different cost-sharing payment schemes. The sum of the percentages is less than 100 because conditions such as psychosis and alcohol or drug problem (<2%) are not shown in the figure.

ED visits increased rapidly in Taiwan, and non-emergent ED visits took a significant portion. There was a 46% increase in annual ED visits from 2000 to 2010 (annual ED visits were 179.0 per 1000 population in 2000 vs. 261.3 per 1000 population in 2010) while the growth rate of annual ED visits dropped down by 77.7% after increasing the cost-sharing payment of ED visits in 2005 (ED growth rate was 35.3% in 2000–2005 vs. 7.9% in 2005–2010). There were apparent differences in numbers, growth rates, and distribution among types of ED visits before and after 2005 (Figure 1).

The introduction of the cost-sharing payment scheme in 2005 had slowed down the increase of ED visits, but its effect varied by the types of ED visits. The share of non-emergent ED visits kept increasing while other types of ED visits gradually decreased their share after 2005. Although there is a clear drop in growth rates of all types of ED visits after 2005 (non-emergent visit: 46.5% vs. 19.4%, emergent visits: 31.7% vs. 4%, injuries: 27% vs. 1.7%), the growth rate of non-emergent visits remained strong (19.4%) (Figure 1).

### 3.2. Emergernt Visits vs. Non-Emergent Visits

There was a substantive difference in systematic-, personal- and ED visit-level factors between emergent visits and non-emergent ED visits. The proportion of non-emergent visits increased with the study year. Females were more like to have non-emergent visits than males. Patients aged between 0 and 19 formed the leading user group for ED and accounted for almost one-third of total ER visits (31.2%, 148,675 visits out of total ED visits), followed by young adults (25.1%) and mid-age (20.1%). Almost half of ED visits occurred in metropolitan hospitals (48.0%), followed by medical centers (27.8%) and local community hospitals (24.2%) (Table 1).

### 3.3. Characteristics of Non-Emergent Visits

Study year, personal demographic factors, socioeconomic factors, personal health utilization history, and visit characteristics were independent factors for non-emergent ED visits. Patients made more and more non-emergent ED visits regardless of the increase in the cost-sharing payment after 2005. (AOR of 2005 = 1.12, AOR of 2010 = 1.30, *p* < 0.001). Females and young age groups were more likely to undertake non-emergent ED visits (*p* < 0.001). Patients with higher incomes, living in more urbanized areas, or in the middle or southern areas of Taiwan also had a higher propensity for non-emergent ED visits (all *p* < 0.001) (Table 2).

Moreover, the regression model showed that patients with a less severe condition might undertake a non-emergent ED visit. Patients who had been hospitalized (AOR of no previous hospitalization = 1.20, *p* < 0.001) or had visited an ED (AOR of no previous ED use = 1.11, *p* < 0.001) in the past 365 days were less likely to have non-emergent ED visits, whereas those who were frequent outpatient users (AOR of high previous OPD use = 1.04, *p* < 0.001) or TCM users (AOR of high previous TCM use = 1.04, *p* < 0.001) were more likely to have non-emergent ED visits (Table 2).

Non-emergent ED visits more likely occurred in metropolitan and local community hospitals compared with medical centers (AOR = 1.14 for a metropolitan hospital, AOR = 1.16 for local community hospital, *p* < 0.001). In addition to clear seasonality and weekday effects (*p* < 0.001), a waived cost-sharing payment also correlated with non-emergent ED visits (*p* < 0.001) (Table 2).

### 3.4. Average Marginal Effects of Factors Associated with Non-Emergent ED Visits

**Figure 2:**
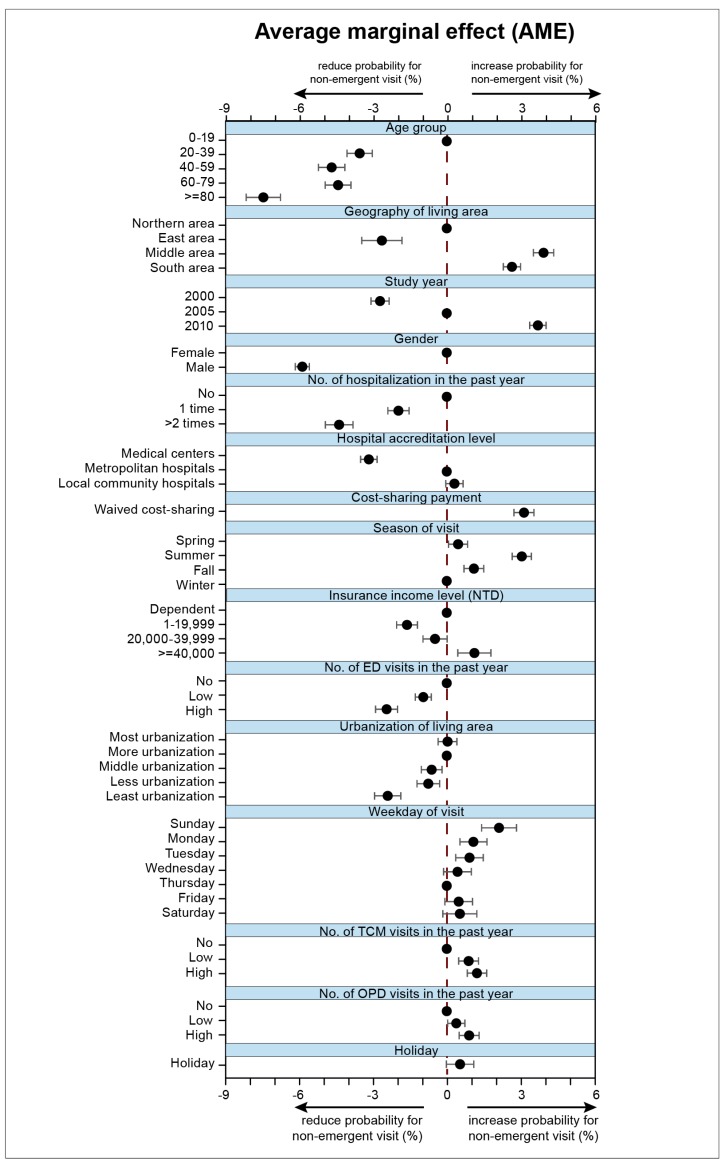
Average marginal effects (AME) of factors for non-emergent ED visits ordered by effect size from three representative study cohorts (LHID2000, LHID2005, LHID2010) in Taiwan. (*n* = 1,000,000 for each cohort, total ED visits, *n* = 475,862). AME were calculated using a logistic model adjusted for listed factors, in addition to Charlson’s index. The most frequent level of each factor was used as the base value (reference line at zero).

To further quantify the effects of factors associated with non-emergent ED visits, AME were calculated and can be interpreted as the maximum effect of each factor on non-emergent ED visits. All factors except holidays were significantly associated with non-emergent ED visits with varied but moderate levels of AME, which suggested that these factors were small but still significant to non-emergent ED visits (Figure 2).

Age was the most influential factor for non-emergent ED visits. On average, being an older adult aged >80 would reduce the probability for non-emergent ED visits by 7.5% (95% C.I. = 8.18–6.78, *p* < 0.001). Following age was geographical residence (AME of living in the eastern area = −2.65, (95% C.I.= −3.47–−1.83%), and AME of living in the middle area = 3.95, (95% C.I. = 3.53–4.36), (both *p* < 0.001) and study year (AME of year 2000 = −2.72%, (95% C.I. = −3.09–−2.35), (AME of year 2010 = 3.72% (95% C.I. = 3.38–4.05), (both *p* < 0.001) were strongly associated with non-emergent ED visits (Supplementary Table S1).

Previous hospitalization history was the most predictive factor among health utilization history factors. For patients who had been admitted previously, the chance of non-emergent visits would decline 1.97% (95% C.I. = 2.40–1.54), *p* < 0.001) and 4.39% (95% C.I. = 4.95–3.83), *p* < 0.001) for those admitted more than one time. (Figure 2, Supplementary Table S1)

Cost-sharing payment also played a role in non-emergent ED visits. Patients seemed sensitive to cost-sharing, so that the eligibility to waive the cost-sharing payment would further drive non-emergent ED visits by 3.15% (95% C.I. = 2.74–3.56%, *p* < 0.001) (Figure 2, Supplementary Table S1).

## 4. Discussion

Increasing numbers of ED visits have posed a challenge to health systems in many countries, and the understanding of non-emergent ED visits has remained limited and contentious. This retrospective study analyzed ED visits using three representative cohorts from routine data to explore the profiles and longitudinal pattern change of non-emergent ED visits in Taiwan. The annual ED visit rate increased up to 261.3 per 1000 population in 2010, and a significant one-third of them were considered non-emergent ED visits. Systematic-, personal-, and ED visit-level factors had contributed to non-emergent ED visits. The introduction of a cost-sharing payment scheme in 2005 slowed down the increase of ED visits, but the growth rate of non-emergent use of ED service remained strong. Additionally, every single personal- and ED visit-level factor was significantly associated with non-emergent ED visits with varied impact. We concluded that non-emergent ED visits were associated with multifaceted factors, while younger age, geography of living area, change of cost-sharing payment, and females were the most predictive factors.

The most challenging part of this study was determining non-emergent ED visits, which were largely estimated, relying on diagnostic criteria or the judgement of clinical staff in previous studies [18,19,20,23,24,25,26,27]. The current study used the NYU algorithm, originally developed and validated in US, which is highly correlated with mortality and admission [24]. Although such a method made it possible to analyze real world data, we had also adjusted the probability threshold for classifying ED visits to better fit Taiwan’s system. More validation studies are required before translating our finding into practice [18]. Moreover, it is also challengeable to classify ED visits relying on the judgement of clinical staff. There is a growing realization that these methods are probably inappropriate as it is the patients who decide where to obtain their medical care and do so on the basis of their judgement as to the urgency of their medical condition [28]. A novel risk-stratified model, i.e. classifying patient according to his own risk of admission and mortality, based on a predict-scoring model rather than any arbitrary criteria should be more suitable for the optimization of ED use [29,30].

However, the current study does add information to understand non-emergent use of ED from the patients’ side. Non-emergent use of ED visits in the real world largely represented the patients’ preference [31,32]. Our result was supported by this point of view. Patients belonging to a higher insurance income level, living in more urban area, or having less illness are more likely to make non-emergent ED visits [12,13]. Moreover, patients are prone to make non-emergent ED visits on Sunday and at the beginning of a week. These finding are compatible with previous studies that suggested nowadays patients usually use ED services just because they feel they need to (e.g., perceived urgency, anxiety, the value of reassurance from ED, and convenience) [12,14], regardless of whether or not they meet the definition of a medical emergency [31,33].

Our study revealed a varied effect of copayment on non-emergent ED visits in the real world. ED visits are very sensitive to copayment. Although the introduction of a copayment for ED visits seemed to cut down ED visits effectively, a higher growth rate for non-emergent visits in the second five years implies that the copayment relatively reduced urgent attendances more than the nonurgent attendances. The finding might be looked at as counterintuitive since copayment is thought to be one of the most effective strategies for non-emergent ED visits previously [34]. However, our finding was supported by price effects studies in Taiwan, which indicated that copayment has little effect on patients with low medical expenditure, high income, and low chronic diseases [21,35,36], which share the same features with patients prone to use non-emergent ED visits. Our finding showed that the copayment strategy could be with consequences and there is a need for more real world data before implementation [2].

Age is the most predictive factor for ED visits. Our study showed that the younger population had shown a strong need for non-emergent ED visits. Lacking of social support, fear, and a desire for immediate medical care always drives parents to use non-emergent ED visits. The findings were compatible with previous studies on other health systems [7,9,10,11]. In fact, not only parents believed that they acted appropriately for non-emergent ED visits, but physicians also approved of their decisions, as an interviewing survey suggested [37]. Additionally, non-emergent ED visits by older people should not be overlooked, as those visits may be associated with fragmented care and emergent admissions [38,39,40]. Interventions toward enhancing health literacy targeted at stakeholders of different age groups is warranted to optimize the utilization of ED services [4,32,41,42,43].

Geographic variation in non-emergent ED visits does exist in Taiwan’s ED care system. Since Taiwan’s NHI had provided ubiquitous and unrestricted health service to every people, the systematic-level difference should be very minimal. While previous studies suggested a link between low spatial density of primary care service and non-emergent ED visits [44,45], some recent studies suggested that fragmented primary care service could be a key reason in addition to spatial access [15,46,47]. Because of a high tendency for non-emergent ED use in the middle and southern Taiwan, field studies focusing on the continuity of localized health services in these area should be considered. 

Our result clearly indicated that the so-called holiday effect does exist, but it is not a strong trajectory compared with other systematic- and personal-level factors. Several previous study showed that patients tend to use non-emergent ED visits in out-of-office hours or holidays [9,48]. Some regulators attempted to extend office-hour service to relieve non-emergent use of ED visits with satisfactory effects [49,50]. Our results showed that the maximum AME of day of the week and holiday is less than 3%, implying that the optimal effect would be less than 3% if the Taiwan government tries to provide seven days health service. A rigorous evaluation using real world data is the necessity for policy making.

Our result showed that non-emergent ED visit is a multifaceted phenomenon, thus requires collaborate efforts from every aspect. Our results clearly showed non-emergent ED visits associated with the cost-sharing payment, personal factors, socioeconomic factors, environmental factors, and characteristics of visits; each of these factors accounted for a small but significant effect on non-emergent use of ED services. Most policymakers have combined one or more of the following strategies to improve ED services: (1) cost sharing; (2) strengthening primary care; (3) pre-hospital triage; (4) coordination; (5) education and self-management support; (6) barriers to access emergency departments [2,51]. However, these strategies are not without consequence, and evidence from routinely collected data was thus a necessity for better ED service [15]. Recently, an innovative population model in an underserved Dallas community reported a limited but convincing result in improving ED services by integrating wellness and other upstream strategies to address social determinants of health [52]. Moreover, meaningful use of routine data from EDs that predict admission in advance may shed new light on the appropriateness of non-emergent ED visits [29,30].

Our results provided compelling evidence for profiles and longitudinal pattern changes of non-emergent ED visits in Taiwan. However, some limitations are worth noting to translate these findings to policy. First, the categorization of “non-emergent” ED visits is very challenging. The current study used the NYU algorithm that is originally developed for US, and showed high readmission and mortality rates in emergent ED visit, but not for “non-emergent” ED visits. Thus a further validation study is needed before applying our study. Third, the disadvantaged population may have less access to ED services. In Taiwan’s national health insurance scheme, everyone should be covered (coverage rate in Taiwan exceeds 99%) and have equal and full access to outpatient services, as well as ED services. Thus, this issue should be minimal in the current study. Fourth, the current study incorporated three waves of cross-sectional data to observe the change of patterns across three cohorts at the national level. Because of a change of data policy in Taiwan, the NHIRD did not provide any data after 2013, and thus the current study was limited and may not be extrapolated to most current study. Moreover, a more sophisticated mixed-effect model should be considered for a more precise estimation of effect of each factors.

## 5. Conclusions

The current study provides compelling evidence for profiles and longitudinal pattern changes of non-emergent ED visits in Taiwan. A significant one-third of ED visits were considered as non-emergent. Non-emergent ED visits were associated with multifaceted factors included the change of cost-sharing, personal-level factors, and ED visit-level factors. Analyzing real world data would add information, enhancing evidence-based strategies. We urge more studies on developing novel risk-stratified policy to optimize ED use.

## Figures and Tables

**Table 1:** Personal and ED visit characteristics by ED visit classification from three representative study cohorts (LHID2000, LHID2005, LHID2010) in Taiwan. (*n* = 1,000,000 for each cohort; total ED visits, *n* = 475,862).

Characteristics	Emergent Visits		Non-Emergent ED Visits		
	*n* = 109,183	(%)	*n* = 208,234	(%)	*p*-Value
Systematic-level factor					<0.001
2000	72,624	(27.1)	49,381	(23.7)	
2005	95,635	(35.7)	72,348	(34.7)	
2010	99,469	(37.2)	86,405	(41.5)	
Demographic factors					
Gender					<0.001
Female	127,003	(47.4)	111,312	(53.5)	
Male	140,719	(52.6)	96,815	(46.5)	
Age group					<0.001
0–19	78,256	(29.2)	70,419	(33.8)	
20–39	67,189	(25.1)	52,477	(25.2)	
40–59	55,280	(20.6)	40,393	(19.4)	
60–79	48,729	(18.2)	33,726	(16.2)	
80–	18,274	(6.8)	11,119	(5.3)	
Comorbidities					<0.001
Charlson‘s index, mean, (SD)	2.8	(6.33)	2.2	(5.76)	
Socioeconomic factors					
Income level (NTD)					<0.001
Dependent	109,087	(40.7)	93,241	(44.8)	
1–19999	89,431	(33.4)	61,322	(29.5)	
20000–39999	50,969	(19.0)	39,293	(18.9)	
40000–	18,241	(6.8)	14,278	(6.9)	
Geographical Residence					<0.001
Northern area	137,985	(51.5)	103,466	(49.7)	
East area	9,771	(3.6)	6,090	(2.9)	
Middle area	45,387	(17.0)	38,390	(18.4)	
Southern area	74,585	(27.9)	60,188	(28.9)	
Urbanization of living area					<0.001
Most urbanization	75,390	(28.2)	58,356	(28.0)	
More urbanization	77,997	(29.1)	61,560	(29.6)	
Middle urbanization	47,178	(17.6)	37,252	(17.9)	
Less urbanization	38,749	(14.5)	30,436	(14.6)	
Least urbanization	28,414	(10.6)	20,530	(9.9)	
Past health utilization in last year					
No. of hospitalizations					<0.001
None	195,207	(72.9)	160,505	(77.1)	
One time	38,841	(14.5)	27,737	(13.3)	
≥ two times	33,680	(12.6)	19,892	(9.6)	
No. of ED visits					<0.001
None	124,897	(46.7)	102,837	(49.4)	
Low (1–2 times)	90,953	(34.0)	70,450	(33.8)	
High (≥3 times)	51,878	(19.4)	34,847	(16.7)	
No. of outpatient visits					<0.001
Low (0-11 times)	96,740	(36.1)	76,866	(36.9)	
Middle (12-26 times)	86,096	(32.2)	68,418	(32.9)	
High (≥ 27 times)	84,892	(31.7)	62,850	(30.2)	
No. of TCM outpatient visits ^1^					<0.001
None	185,426	(69.3)	141,928	(68.2)	
Low (1–2 times)	39,721	(14.8)	31,578	(15.2)	
High (≥ 3 times)	42,581	(15.9)	34,628	(16.6)	
Hospital accreditation level					<0.001
Medical centers	36,954	(13.8)	95,237	(45.8)	
Metropolitan hospitals	50,656	(18.9)	177,672	(85.4)	
Local community hospitals	21,573	(8.1)	93,770	(45.1)	
Season					<0.001
Spring (Mar–May)	69,444	(25.9)	52,606	(25.3)	
Summer (Jun–Aug)	66,096	(24.7)	55,362	(26.6)	
Fall (Sep–Nov)	60,918	(22.8)	46,896	(22.5)	
Winter (Dec–Feb)	71,270	(26.6)	53,270	(25.6)	
Public holiday					<0.001
No (781 days)	168,376	(62.9)	126,754	(60.9)	
Yes (314 days)	99,352	(37.1)	81,380	(39.1)	
Day of the week					<0.001
Monday	36,650	(13.7)	28,267	(13.6)	
Tuesday	33,885	(12.7)	25,950	(12.5)	
Wednesday	33,222	(12.4)	24,966	(12.0)	
Thursday	33,461	(12.5)	24,562	(11.8)	
Friday	33,582	(12.5)	25,178	(12.1)	
Saturday	40,349	(15.1)	31,139	(15.0)	
Sunday	56,579	(21.1)	48,072	(23.1)	
Cost-sharing payment					<0.001
Yes	221,614	(82.8)	170,475	(81.9)	
Waived	46,114	(17.2)	37,659	(18.1)	

^1^ TCM: Traditional Chinese Medicine was considered a mainstream visit in addition to outpatient clinics and was covered in Taiwan’s insurance scheme.

**Table 2:** Adjusted odds ratios (AOR) for non-emergent ED visits from three representative study cohorts (LHID2000, LHID2005, LHID2010) in Taiwan. (*n* = 1,000,000 for each cohort; total ED visits, *n* = 475,862).

Characteristics	Adjusted Odds Ratios			
	AOR	(95 % C.I.)	*p*-Value	Sig. ^1^
Systematic-level factor				
2000		-ref-		
2005	1.12	(1.10–1.14)	<0.001	***
2010	1.30	(1.28–1.32)	<0.001	***
Personal-level factors				
Demographic factors				
Gender				
Female	1.27	(1.26–1.29)	<0.001	***
Male		-ref-		
Age group				
0-19	1.36	(1.32–1.40)	<0.001	***
20-39	1.18	(1.15–1.21)	<0.001	***
40-59	1.12	(1.09–1.16)	<0.001	***
60-79	1.14	(1.11–1.17)	<0.001	***
80-		-ref-		
Comorbidities				
Charlson‘s index	1.00	(1.00–1.00)	<0.001	***
Socioeconomic factors				
Income level (NTD)				
Dependent	1.07	(1.05–1.09)	<0.001	***
1–19999		-ref-		
20000–39999	1.05	(1.03–1.07)	<0.001	***
40000–	1.12	(1.09–1.15)	<0.001	***
Geographical Residence				
Northern area	1.12	(1.08–1.16)	<0.001	***
East area		-ref-		
Middle area	1.31	(1.27–1.36)	<0.001	***
Southern area	1.25	(1.20–1.29)	<0.001	***
Urbanization of living area				
Most urbanization	1.11	(1.08–1.13)	<0.001	***
More urbanization	1.11	(1.08–1.13)	<0.001	***
Middle urbanization	1.08	(1.05–1.10)	<0.001	***
Less urbanization	1.07	(1.05–1.10)	<0.001	***
Least urbanization		-ref-		
Past health utilization in last year				
No. of hospitalizations				
None	1.20	(1.17–1.23)	<0.001	***
One time	1.11	(1.08–1.13)	<0.001	***
≥ two times		-ref-		
No. of ED visits				
None	1.11	(1.09–1.13)	<0.001	***
Low (1–2 times)	1.06	(1.04–1.08)	<0.001	***
High (≥3 times)		-ref-		
No. of outpatient visits				
Low (0–11 times)		-ref-		
Middle (12–26 times)	1.02	(1.00–1.03)	0.03	*
High (≥ 27 times)	1.04	(1.02–1.06)	<0.001	***
No. of TCM outpatient visits ^2^				
None		-ref-		
Low (1–2 times)	1.04	(1.02–1.05)	<0.001	***
High (≥ 3 times)	1.05	(1.04–1.07)	<0.001	***
ED visit-level factors				
Hospital accreditation level				
Medical centers		-ref-		
Metropolitan hospitals	1.14	(1.12–1.16)	<0.001	***
Local community hospitals	1.16	(1.14–1.17)	<0.001	***
Season				
Spring (Mar–May)	1.02	(1.00–1.04)	0.02	*
Summer (Jun–Aug)	1.13	(1.12–1.15)	<0.001	***
Fall (Sep–Nov)	1.05	(1.03–1.06)	<0.001	***
Winter (Dec–Feb)		-ref-		
Public holiday				
No		-ref-		
Yes	1.02	(1.02–1.05)	0.056	
Day of the Week				
Monday	1.05	(1.02–1.07)	<0.001	***
Tuesday	1.04	(1.02–1.06)	0.001	**
Wednesday	1.02	(1.02–1.06)	0.129	
Thursday		-ref-		
Friday	1.02	(1.00–1.04)	0.091	
Saturday	1.02	(0.99–1.05)	0.131	
Sunday	1.09	(1.06–1.12)	<0.001	***
Cost-sharing payment				
Yes		-ref-		
Waived	1.14	(1.12–1.16)	<0.001	***

^1^ Significance level: *: *p* < 0.05; **: *p* < 0.01; ***: *p* < 0.001. ^2^ TCM: Traditional Chinese Medicine was considered a mainstream visit in addition to outpatient clinics and was covered in Taiwan’s insurance scheme.

## Supplementary Materials

The following are available online at https://www.mdpi.com/1660-4601/16/11/1999/s1, Table S1: Average marginal effect (AME) by factors for non-emergent ED visits from three representative study cohorts (LHID2000, LHID2005, LHID2010) in Taiwan. (*n* = 1,000,000 for each cohort, total ED visits, *n* = 475,862).
