# Supplementary material for: Characteristics of Non-Emergent Visits in Emergency Departments: Profiles and Longitudinal Pattern Changes in Taiwan, 2000–2010
Source: Int J Environ Res Public Health. 2019 Jun 5;16(11):1999. doi: 10.3390/ijerph16111999 (PMC6603954; doi:10.3390/ijerph16111999)
Supplement: Supplementary file 1 [file ijerph-16-01999-s001.pdf]

International Journal of
Environmental Research and Public Health

MDPI

Supplementary Material

Characteristics of Non-Emergent Visits in Emergency Departments: Profiles and Longitudinal Pattern Changes in Taiwan, 2000–2010

Liang-Chung Huang, Wu-Fu Chung, Shih-Wei Liu, Jau-Ching Wu, Li-Fu Chen and Yu-Chun Chen

Table S1. Average marginal effect (AME) by factors for non-emergent ED visits from three representative study cohorts (LHID2000, LHID2005, LHID2010) in Taiwan. (n = 1,000,000 for each cohort, total ED visits, n = 475,862).

	Average marginal effect (AME)[1]			
	AME	(95 % C.I.)	P-value	Sig.[2]
**Systematic-level factor**				
2000	-2.72	(-3.09--2.35)	<0.001	***
2005		-base level-		
2010	3.72	(3.38-4.05)	<0.001	***
**Personal-level factors**				
**Demographic factor**				
Gender				
Female		-base level-		
Male	-5.89	(-6.18--5.60)	<0.001	
Age group				
0-19		-base level-		
20-39	-3.55	(-4.07--3.04)	<0.001	***
40-59	-4.69	(-5.23--4.16)	<0.001	***
60-79	-4.43	(-4.96--3.91)	<0.001	***
80-	-7.48	(-8.18--6.78)	<0.001	***
**Socio-economic factors**				
Income (NTD)				
Dependent		-base level-		
1-19999	-1.62	(-2.05--1.19)	<0.001	***
20000-39999	-0.48	(-0.97-0.01)	0.057	
40000-	1.13	(0.45-1.80)	0.001	**
Geographical Residence				
Northern area		-base level-		
Eastern area	-2.65	(-3.47- -1.83)	<0.001	***
Middle area	3.95	(3.53-4.36)	<0.001	***
Southern area	2.66	(2.31-3.01)	<0.001	***
Urbanization of living area				

Most urbanization	0.03	(-0.35-0.41)	0.871	
More urbanization		-base level-		
Middle urbanization	-0.61	(-1.04--0.19)	0.005	**
Less urbanization	-0.75	(-1.21--0.29)	0.001	**
Least urbanization	-2.41	(-2.94--1.87)	<0.001	***
**Past health utilization in last year**				
Any hospitalization				
None		-base level-		
One time	-1.97	(-2.40--1.54)	<0.001	***
>= two times	-4.39	(-4.95--3.83)	<0.001	***
No. of ED visits				
None		-base level-		
Low (1-2 times)	-0.96	(-1.28--0.63)	<0.001	***
High (>=3 times)	-2.45	(-2.90--2.01)	<0.001	***
No. of outpatient visits				
Low (0-11 times)		-base level-		
Middle (12-26 times)	0.39	(0.04-0.74)	0.03	*
High (>= 27 times)	0.91	(0.51-1.32)	<0.001	***
No. of TCM[3] outpatient visits				
None		-base level-		
Low (1-2 times)	0.89	(0.49-1.30)	<0.001	***
High (>= 3 times)	1.23	(0.84-1.63)	<0.001	***
**ED visit-level factors**				
Hospital accreditation level				
Medical centers	-3.18	(-3.52--2.84)	<0.001	***
Metropolitan hospitals		-base level-		
Local community hospitals	0.31	(-0.04-0.67)	0.085	
Season				
Spring (Mar-May)	0.46	(0.07-0.85)	0.02	*
Summer (Jun-Aug)	3.06	(2.67-3.45)	<0.001	***
Fall (Sep-Nov)	1.11	(0.71-1.51)	<0.001	***
Winter (Dec-Feb)		-base level-		
Holiday				
No		-base level-		
Yes	0.54	(-0.01-1.10)	0.056	
Day of the Week				
Sunday	2.13	(1.42-2.85)	<0.001	***
Monday	1.09	(0.54-1.64)	<0.001	***
Tuesday	0.93	(0.37-1.49)	0.001	**
Wednesday	0.44	(-0.13-1.00)	0.129	
Thursday		-base level-		
Friday	0.49	(-0.08-1.05)	0.091	

Saturday	0.53	(-0.16-1.23)	0.131	
Cost-sharing				
Yes		-base level-		
Waived	3.15	(2.74-3.56)	<0.001	***

[1] AME were calculated using a logistic model adjusted for listed factors in addition to Charlson's index. The most frequent level of each factor was used as the base value. [2] Significance level: *: P<0.05; **: P<0.01; ***: P<0.001. [3] TCM: Traditional Chinese Medicine was considered a mainstream visit in addition to outpatient clinics and covered in Taiwan's insurance scheme.

© 2019 by the authors. Submitted for possible open access publication under the terms and conditions of the Creative Commons Attribution (CC BY) license (http://creativecommons.org/licenses/by/4.0/).
